# Prevalence and Antimicrobial Resistance Profiles of *E. coli*, *P. mirabilis*, and *E. cloacae* Complex Isolated from Dogs with Otitis Externa

**DOI:** 10.3390/antibiotics15040343

**Published:** 2026-03-27

**Authors:** Ionela Popa, Ionica Iancu, Alexandru Gligor, Kalman Imre, Emil Tîrziu, Timea Bochiș, Călin Pop, Janos Degi, Andrei Ivan, Michael Dahma, Ana-Maria Plotuna, Sebastian Alexandru Popa, Marius Pentea, Viorel Herman, Ileana Nichita

**Affiliations:** 1 Department of Semiology, Faculty of Veterinary Medicine, University of Life Sciences “King Mihai I” from Timisoara, 300645 Timisoara, Romania; ionela.popa@usvt.ro (I.P.); timea.bochis@usvt.ro (T.B.); calinpop@usvt.ro (C.P.); 2 Department of Infectious Diseases and Preventive Medicine, Faculty of Veterinary Medicine, University of Life Sciences “King Mihai I” from Timisoara, 300645 Timisoara, Romania; alexandru.gligor@usvt.ro (A.G.); janosdegi@usvt.ro (J.D.); viorel.herman@fmvt.ro (V.H.); 3 Department of Food Safety and Hygiene, Faculty of Veterinary Medicine, University of Life Sciences “King Mihai I” from Timisoara, 300645 Timisoara, Romania; kalmanimre@usvt.ro (K.I.); sebastian.popa@usvt.ro (S.A.P.); 4 Department of Microbiology, Faculty of Veterinary Medicine, University of Life Sciences “King Mihai I” from Timisoara, 300645 Timisoara, Romania; emiltirziu@usvt.ro (E.T.); andreialexandru.ivan.fmv@usvt.ro (A.I.); michael.dahma@usvt.ro (M.D.); ileananichita@usvt.ro (I.N.); 5 Department of Animal Nutrition, University of Life Sciences “King Mihai I” from Timisoara, 300645 Timisoara, Romania; anamaria.plotuna@usvt.ro; 6 Department of Anatomy, University of Life Sciences “King Mihai I” from Timisoara, 300645 Timisoara, Romania; mariuspentea@usvt.ro; 7 Academy of Romanian Scientists (AOSR), Splaiul Independentei 54, 050094 Bucharest, Romania

**Keywords:** AMR, MDR, canine otitis externa, *E. coli*, *P. mirabilis*, *E. cloacae* complex, ESBL, AmpC

## Abstract

**Background/Objectives:** Antimicrobial resistance (AMR) in companion animals is an emerging public health threat due to zoonotic potential and limited therapeutic options. Dogs with otitis externa may harbor multidrug-resistant (MDR) bacteria, including *Escherichia coli* (*E. coli*), *Proteus mirabilis* (*P. mirabilis*), and *Enterobacter cloacae* complex (*E. cloacae* complex), some producing extended-spectrum beta-lactamase (ESBL) or AmpC β-lactamases. This study aimed to assess the prevalence, AMR patterns, MDR occurrence, β-lactamase production, and co-infection profiles of these pathogens in canine otitis externa. **Methods:** Ear canal samples were collected from 592 dogs presenting clinical signs of otitis externa, with one sample per dog included in the analysis. Samples were collected from veterinary clinics in Timiș County, Romania, from 2022 to 2025. Samples were cultured on blood agar and MacConkey agar, followed by biochemical testing and MALDI-TOF mass spectrometry for bacterial identification. Antimicrobial susceptibility testing against 15 agents across six classes was performed using the VITEK^®^ 2 system. MDR and β-lactamase production (ESBL, AmpC) were determined according to CLSI 2018 veterinary guidelines. Co-isolation with bacterial and fungal species were recorded. **Results:** *E. coli*, *P. mirabilis*, and *E. cloacae* complex were isolated in 9.12%, 6.25%, and 1.2% of cases, respectively. *E. coli* exhibited the highest resistance to aminoglycosides (tobramycin 72.2%, gentamicin 61.1%) and full susceptibility to carbapenems. *P. mirabilis* showed the highest resistance to ampicillin (54%) and trimethoprim + sulfamethoxazole (46%), with complete susceptibility to carbapenems and fluoroquinolones. *E. cloacae* complex displayed universal resistance to cephalosporins but remained susceptible to non-cephalosporin β-lactams (piperacillin–tazobactam), carbapenems and aminoglycosides. MDR prevalence was 35.2% for *E. coli*, 18.9% for *P. mirabilis*, and 14.3% for the *E. cloacae* complex. ESBL production was detected in 13% of *E. coli* and 8.1% of *P. mirabilis* isolates, while all *E. cloacae* complex isolates were AmpC-positive. Co-isolations were common, primarily involving *Staphylococcus pseudintermedius* (*S. pseudintermedius*) and *Malassezia pachydermatis* (*M. pachydermatis*). **Conclusions:** MDR and β-lactamase-producing bacteria were identified in dogs with otitis externa, emphasizing the importance of routine antimicrobial susceptibility testing, targeted therapy based on local resistance profiles, and continuous AMR surveillance to prevent treatment failure and mitigate zoonotic risk.

## 1. Introduction

Antimicrobial resistance (AMR) constitutes a major public health challenge on a global scale, representing one of the most pressing threats to contemporary healthcare systems [1,2,3,4,5,6,7]. Sustained antibiotic exposure is a key factor in the acquisition of resistance mechanisms by *Escherichia coli* (*E. coli*). Consequently, animals harboring antibiotic-resistant bacteria, such as *E. coli*, may constitute important reservoirs for transmission, infection, and colonization in humans [1].

Dogs living in close contact with their owners frequently interact with shared household surfaces, creating a significant pathway through which multidrug-resistant (MDR) bacteria disseminate between species [8,9]. In addition, advances in veterinary medicine, together with the growing societal commitment to companion animal welfare, have contributed to prolonged survival in pets. This increased longevity has led to a higher proportion of geriatric animals requiring antimicrobial treatment, as they commonly present with chronic disorders or conditions associated with impaired immune function [8]. Notably, bacteria possess the capacity to transfer genetic cassettes that encode resistance to multiple antimicrobial classes, and recent years have seen an increase in reports of resistance dissemination [9].

*E. coli* represents a bacterium with substantial biological diversity, including strains that function as harmless members of the intestinal microbiota as well as variants capable of causing intraintestinal or extraintestinal infections [10,11]. Commensal *E. coli* is recognized as one of the earliest bacterial species to colonize the gastrointestinal tract, establishing itself immediately after birth [10,12].

The global increase in bacteria that produce extended-spectrum beta-lactamase (ESBL), especially *E. coli*, in humans and companion animals may reduce the effectiveness of these essential antimicrobial agents. Moreover, the simultaneous occurrence of ESBL expression and resistance to additional classes of antibiotics can further restrict available treatment options for bacterial infections [13,14]. ESBL-mediated resistance is frequently linked to reduced susceptibility to fluoroquinolones, aminoglycosides, and sulfamethoxazole/trimethoprim, all of which are considered critically important for human clinical use [15].

The bacterium *Proteus mirabilis* (*P. mirabilis*), characterized as a Gram-negative rod, is widely recognized for its involvement in opportunistic infections affecting human hosts. Taxonomically, it is placed within the order *Enterobacterales* and assigned to the family *Morganellaceae.* This species is capable of persisting in diverse environmental reservoirs as well as colonizing the vertebrate host’s gastrointestinal tract [16]. This species is notably implicated in urinary tract infections, particularly among patients with prolonged indwelling catheters, and is also associated with ear infections, burn-associated infections, diarrhea, and keratitis, as well as septicemia [17,18]. Beyond its clinical manifestations, *P. mirabilis* serves as a reservoir of genes related to virulence and antibiotic resistance, raising significant public health concerns. The species exhibits notable antibiotic resistance, reflecting its capacity to tolerate commonly used antibiotics [18].

Members of the *Enterobacter cloacae* complex (*E. cloacae* complex) are recognized as significant opportunistic pathogens affecting both human and animal hosts [19]. *E. cloacae* consists of Gram-negative bacterial organisms classified within the family Enterobacteriaceae [20].

*Enterobacter cloacae* (*E. cloacae*) is regarded as the species of greatest clinical relevance within its genus, being frequently implicated in hospital-acquired infections in humans. Among companion animals, infections attributed to this organism are comparatively uncommon; however, when present, they may involve urinary tract infections, pneumonia, otitis externa, wound infections, peritonitis, infections at intravenous catheter insertion sites, and dermatitis [21].

The worldwide increase in resistance to antimicrobial agents within the genus *Enterobacter* represents a major concern in human healthcare settings. This trend contributes to a greater probability of therapeutic failure following antibiotic administration in both human and veterinary patients. Furthermore, resistant bacterial strains identified in companion animals may constitute a public health risk if transmission to humans occurs through close interaction with pets. Therefore, evaluating the frequency and distribution of resistant *Enterobacter* isolates is essential from both veterinary and public health standpoints [21].

In the context of otitis externa in companion animals, topical interventions frequently provide effective short-term relief; however, repeated inflammatory or infectious events can evolve into chronic otitis externa. The chronic form is typified by morphological modifications of the ear canal, ongoing discomfort, and diminished therapeutic responsiveness. Inadequate identification and management of the primary etiology often lead to extended antimicrobial administration, thereby facilitating the emergence and expansion of bacterial populations exhibiting antimicrobial resistance [22].

The aim of this study was to assess the prevalence, antimicrobial resistance profiles, MDR occurrence, and β-lactamase production, such as ESBL and AmpC (AmpC β-lactamase), of *E. coli*, *P. mirabilis*, and the *E. cloacae* complex isolated from dogs with otitis externa. Additionally, the study sought to characterize co-infection patterns associated with these bacteria, providing insights relevant to veterinary clinical practice and the evaluation of potential zoonotic transmission, while informing prudent antimicrobial stewardship.

## 2. Results

### 2.1. Prevalence of Isolated Bacteria

The prevalence of bacterial isolates among the 592 dogs was as follows: *E. coli* 9.12% (*n* = 54), *P. mirabilis* 6.25% (*n* = 37), and the *E. cloacae* complex 1.2% (*n* = 7).

### 2.2. Antimicrobial Resistance

Regarding antimicrobial resistance, *E. coli* exhibited the highest resistance to tobramycin at 72.2% (*n* = 39), followed by gentamicin at 61.1% (*n* = 33). No resistance was observed against amoxicillin + clavulanic acid, piperacillin + tazobactam, cefoxitin, imipenem, and meropenem, with susceptibility reaching 100% (*n* = 54) (Table 1).

*P. mirabilis* demonstrated the highest resistance to ampicillin at 54.0% (*n* = 20), followed by trimethoprim + sulfamethoxazole at 46.0% (*n* = 17). No resistance was observed against amoxicillin + clavulanic acid, piperacillin + tazobactam, cefoxitin, imipenem, meropenem, and ciprofloxacin, with 100% susceptibility (*n* = 37) (Table 1).

The *E. cloacae* complex exhibited 100% resistance (*n* = 7) to cephalothin, cefoxitin, and cefpodoxime. Resistance to cefuroxime and trimethoprim + sulfamethoxazole was 42.9% (*n* = 3), while resistance to ciprofloxacin was 14.3% (*n* = 1). Complete susceptibility (100%, *n* = 7) was observed for piperacillin + tazobactam, cefotaxime, ceftazidime, imipenem, meropenem, gentamicin, and tobramycin (Table 1). This pattern is consistent with inducible chromosomal AmpC β-lactamase activity, which preferentially affects early-generation cephalosporins and cephamycins.

At the class level, *E. coli* exhibited the highest resistance to aminoglycosides at 72.2% [59.2–82.4] (*n* = 39), whereas no resistance was observed to carbapenems, with 100% susceptibility (*n* = 54) (Table 2 and Table 6). *P. mirabilis* showed the highest resistance to β-lactams at 54.0% (*n* = 20), with full susceptibility to carbapenems and fluoroquinolones (100%, *n* = 37) (Table 2). The *E. cloacae* complex demonstrated complete resistance to cephalosporins, 100% [64.6–100] (*n* = 7), but remained fully susceptible to non-cephalosporin β-lactams (piperacillin–tazobactam), carbapenems, and aminoglycosides (100%, *n* = 7) (Table 2 and Table 6).

### 2.3. MDR and β-Lactamase Production

Among *E. coli* strains, 35.2% [24.0–48.3] (*n* = 19) were classified as MDR, and 13.0% [6.5–24.6] (*n* = 7) were identified as ESBL producers (Table 3 and Table 6). All ESBL-producing strains concurrently exhibited resistance to multiple antimicrobial classes and were included in the MDR category (Table 3).

For *P. mirabilis*, 18.9% [9.5–33.3] (*n* = 7) of strains were MDR, while 8.1% [2.8–21.3] (*n* = 3) were ESBL producers. All ESBL-positive strains showed simultaneous resistance to multiple antimicrobial classes and were categorized as MDR (Table 4 and Table 6).

In the *E. cloacae* complex, a single isolate (*n* = 1, 14.3% [2.6–51.3]) fulfilled the criteria for MDR, defined independently of intrinsic AmpC expression. All *E. cloacae* complex isolates (*n* = 7, 100% [64.6–100]) displayed phenotypic resistance patterns consistent with inducible chromosomal AmpC β-lactamase production, which is intrinsic to this species complex (Table 5 and Table 6). This finding reflects intrinsic chromosomal AmpC expression characteristic of the *E. cloacae* complex and should not be interpreted as acquired resistance.

### 2.4. Comparative Statistical Analysis

A comparative statistical evaluation of antimicrobial resistance patterns among *E. coli*, *P. mirabilis*, and *E. cloacae* complex isolates is presented in Table 6. Significant interspecies differences were observed for ampicillin resistance, cephalosporin resistance, aminoglycoside resistance, and fluoroquinolone resistance (*p* < 0.05). No statistically significant differences were identified for trimethoprim + sulfamethoxazole resistance or ESBL production.

Although MDR prevalence appeared higher in *E. coli* compared to *P. mirabilis* and the *E. cloacae* complex, the overall interspecies difference was not statistically significant (χ^2^ = 3.57, df = 2, *p* = 0.17).

Interpretation of results involving the *E. cloacae* complex should be approached with caution due to the limited sample size.

**Table 6:** Statistical analysis of antimicrobial resistance among *E. coli*, *P. mirabilis*, and *E. cloacae* complex isolates from canine otitis externa.

Variable	*E. coli* (*n* = 54) % [95% CI]	*P. mirabilis* (*n* = 37) % [95% CI]	*E. cloacae* Complex (*n* = 7) % [95% CI]	*p*-Value *
Beta-lactams resistance	18.5 [10.4–30.9]	54.0 [38.7–68.6]	NA	<0.001
Cephalosporin resistance	38.9 [27.1–52.1]	8.1 [2.8–21.3]	100 [64.6–100]	<0.001
Aminoglycoside resistance	72.2 [59.2–82.4]	16.2 [7.6–31.6]	0 [0–35.4]	<0.001
Fluoroquinolone resistance	31.5 [20.8–44.4]	0 [0–9.5]	14.3 [2.6–51.3]	0.002
Trimethoprim–sulfamethoxazole resistance	35.2 [24.0–48.3]	46.0 [31.4–61.3]	42.9 [15.8–75.0]	0.62
Carbapenem resistance	0 [0–6.6]	0 [0–9.5]	0 [0–35.4]	–
MDR	35.2 [24.0–48.3]	18.9 [9.5–33.3]	14.3 [2.6–51.3]	0.17
ESBL production	13.0 [6.5–24.6]	8.1 [2.8–21.3]	–	0.48
AmpC phenotype	–	–	100 [64.6–100]	–

* Chi-square or Fisher’s exact test.

### 2.5. Co-Isolation of Microbial Species

Regarding co-isolation, *E. coli* was isolated as a sole pathogen in 11.1% of cases, whereas in the majority of cases (88.9%) it was identified in association with other microorganisms. The most frequently co-isolated species were *S. pseudintermedius* (*n* = 26; 48.2%) and *M. pachydermatis* (*n* = 15; 27.8%) (Table 7).

*P. mirabilis* was recovered as a single agent in 13.5% of cases and co-isolated with other microorganisms in 86.5% of cases. The most common co-isolated species were *S. pseudintermedius* (*n* = 14; 37.8%), *M. pachydermatis* (*n* = 10; 27.0%), and *P. aeruginosa* (*n* = 8; 21.6%) (Table 8).

*E. cloacae* complex was isolated as a sole pathogen in 28.6% of cases and co-isolated in 71.4% of cases. The most frequently co-isolated species were *S. pseudintermedius* (*n* = 2; 28.6%) and *M. pachydermatis* (*n* = 2; 28.6%) (Table 9).

## 3. Discussion

### 3.1. Prevalence and Epidemiological Context

The present study provides regional data on AMR patterns among *E. coli*, *P. mirabilis*, and the *E. cloacae* complex isolated from dogs with otitis externa in Timiș County, Romania.

In the present study, the prevalence of *E. coli* was 9.12%, which differs from the findings reported by De Martino et al. [23] (4.2%) and Bugden [24] (4.2%). Our reported prevalence also exceeds those described by Zamankhan Malayeri et al. [25], who documented a rate of 1.09%, and by Prošić et al. [26], who reported a prevalence of 1.90%. Conversely, our results are comparable to those obtained by Lyskova et al. [27] (10.3%), Petrov et al. [28] (11%) and Niaraki et al. [29] (13.3%).

With regard to *P. mirabilis*, the prevalence identified in our cohort was 6.25%, closely aligning with the findings of De Martino et al. [23], who reported a rate of 6.3%. However, our reported prevalence is higher than those documented by Zamankhan Malayeri et al. [25] (3.26%) and Niaraki et al. [29] (2.7%), while remaining lower than the prevalence reported by Prošić et al. [26] (14.29%).

The relatively low prevalence of the *E. cloacae* complex in this study was 1.2%, which is consistent with its opportunistic role in companion animal infections [30]. However, despite its lower frequency, the presence of inducible AmpC β-lactamases [31] warrants careful clinical attention due to potential therapeutic implications.

These inter-study discrepancies may be attributed to differences in geographic distribution, study populations, sampling strategies, diagnostic methodologies, and temporal variability in antimicrobial resistance patterns, all of which can substantially influence reported prevalence rates.

### 3.2. Resistance to Penicillins

Beta-lactam antibiotics represent a class of broad-spectrum agents commonly utilized in the management of infections affecting both humans and animals. The principal mechanism underlying bacterial resistance to these drugs involves the synthesis of beta-lactamase enzymes [13].

Regarding antimicrobial resistance to penicillins, *E. coli* in the present study exhibited resistance to ampicillin in 18.5% of isolates, which is markedly lower than the 47.7% resistance reported by Rosales et al. [32]. In contrast, no resistance to amoxicillin-clavulanic acid was observed in our isolates, differing from the findings of Rosales et al. [32], who reported a resistance rate of 6.8%, and from Petrov et al. [28], who documented a rate of 32%.

Regarding antimicrobial resistance in *P. mirabilis*, 54% of isolates in the present study exhibited resistance to ampicillin, which is comparable to the rates reported by Kwon et al. [33] (59%) and Rosales et al. [32] (60%). In contrast, no resistance to amoxicillin-clavulanic acid was observed in our isolates (0%), differing from the findings of Rosales et al. [32] (29.2%), Petrov et al. [28] (30%), and Kwon et al. [33] (63%).

These discrepancies may be explained by geographic variation, local prescribing practices, and methodological differences in susceptibility testing.

### 3.3. Resistance to Cephalosporins

In terms of resistance to cephalosporins, *E. coli* in the present study exhibited 13% resistance to cefpodoxime, which is comparable to the 16.3% reported by Rosales et al. [32]. Regarding ceftazidime, 13% of our isolates were resistant, a rate similar to that reported by Rosales et al. [32] (14%). These findings suggest a relatively low level of resistance to third-generation cephalosporins among *E. coli* isolates in this canine population, consistent with previous reports.

In terms of resistance to cephalosporins, *P. mirabilis* in the present study exhibited 8.1% resistance to cefpodoxime, which is lower than the 21.7% reported by Rosales et al. [32]. Regarding ceftazidime, 8.1% of our isolates were resistant, a rate similar to that reported by Rosales et al. [32] (6.5%) but markedly lower than the 50% reported by Kwon et al. [33]. These results indicate a relatively low prevalence of resistance to third-generation cephalosporins among *P. mirabilis* isolates in this canine population, although significant variability is observed across different geographic regions and study settings.

The observed susceptibility to third-generation cephalosporins (cefotaxime and ceftazidime) in the *E. cloacae* complex, despite resistance to cephalothin, cefoxitin, and cefpodoxime, is consistent with inducible AmpC expression, which may not fully compromise extended-spectrum cephalosporins unless derepressed.

### 3.4. Resistance to Carbapenems

In terms of resistance to carbapenems, *E. coli* in the present study exhibited no resistance to imipenem, with all isolates being fully susceptible (100%), a finding consistent with Rosales et al. [32], who also reported 100% susceptibility. These results indicate that carbapenems remain highly effective against *E. coli* isolates from canine otitis externa.

*P. mirabilis* in the present study exhibited no resistance to imipenem (0%), differing from the findings of Rosales et al. [32], who reported a resistance rate of 19.6%. These results suggest that imipenem remains highly effective against *P. mirabilis* isolates from canine otitis externa.

### 3.5. Resistance to Aminoglycosides

Aminoglycoside resistance among *E. coli* isolates in the present study was 61.1% for gentamicin, differing from the 18% and 11.4% reported by Petrov et al. [28] and Rosales et al. [32], respectively. Resistance to tobramycin was 72.2%, substantially higher than the 5% reported by Petrov et al. [28]. These findings indicate a high level of aminoglycoside resistance among *E. coli* isolates from canine otitis externa, highlighting the need for careful antimicrobial selection.

*P. mirabilis* isolates in the present study exhibited 16.2% resistance to both gentamicin and tobramycin. Gentamicin resistance differed from the 6.3%, 28%, and 75% reported by Rosales et al. [32], Petrov et al. [28], and Kwon et al. [33], respectively. Resistance to tobramycin was also 16.2%, contrasting with the 0% reported by Petrov et al. [28]. These results indicate a moderate level of aminoglycoside resistance among *P. mirabilis* isolates, with notable variation across different studies, highlighting the importance of local antimicrobial susceptibility testing.

### 3.6. Resistance to Fluoroquinolones

Fluoroquinolones are among the antibiotics frequently employed in both human and veterinary medical practice, with ciprofloxacin representing the most widely used compound within this class at the global level. Consequently, resistance to fluoroquinolones has been documented in bacterial populations affecting both human and animal hosts [34].

*E. coli* isolates in the present study exhibited 31.5% resistance to ciprofloxacin, which is higher than the 15.9% reported by Rosales et al. [32]. This finding indicates a moderate level of fluoroquinolone resistance among *E. coli* isolates from canine otitis externa, underlining the importance of careful antimicrobial selection.

*P. mirabilis* isolates in the present study exhibited no resistance to ciprofloxacin (0%), differing from the 21.7% and 53% resistance rates reported by Rosales et al. [32] and Kwon et al. [33], respectively. These results indicate that ciprofloxacin remains highly effective against *P. mirabilis* isolates from canine otitis externa, although resistance levels may vary across different populations [32,33].

### 3.7. Resistance to Sulfonamides + Pyrimidines

*E. coli* isolates in the present study exhibited 35.2% resistance to sulfamethoxazole + trimethoprim, which is higher than the 23.3% reported by Rosales et al. [32]. This finding indicates a moderate level of resistance to the sulfonamide + trimethoprim combination among *E. coli* isolates from canine otitis externa, underscoring the importance of careful antimicrobial selection.

*P. mirabilis* isolates in the present study exhibited 46.0% resistance to sulfamethoxazole + trimethoprim, differing from the 16.7% and 72% reported by Rosales et al. [32] and Kwon et al. [33], respectively. These results indicate a moderate level of resistance among *P. mirabilis* isolates, with notable variability across different populations, highlighting the importance of local antimicrobial susceptibility testing.

### 3.8. Multidrug Resistance and ESBL Production

Extensive antibiotic administration has substantially contributed to the emergence of *E. coli* strains exhibiting MDR, recovered from companion animals [35].

In the present study, 35.2% of *E. coli* isolates were classified as MDR, differing from the 24.6% and 47.7% reported by Garcias et al. [36] and Rosales et al. [32], respectively. MDR *P. mirabilis* isolates were observed at a rate of 18.9%, lower than the 52.1% reported by Rosales et al. [32]. Additionally, 13.0% of *E. coli* isolates were ESBL-positive, markedly lower than the 50% reported by Saraiva et al. [22]. These findings indicate a substantial prevalence of MDR in both *E. coli* and *P. mirabilis*, with ESBL production detected in both species, highlighting the importance of ongoing antimicrobial surveillance and prudent therapeutic strategies.

It is important to distinguish between intrinsic chromosomal AmpC expression, which is characteristic of the *E. cloacae* complex, and acquired resistance mechanisms. In this study, all isolates exhibited phenotypic patterns consistent with intrinsic AmpC production; however, only one isolate met the criteria for MDR.

### 3.9. Co-Isolation

The elevated prevalence of polymicrobial infections carries substantial clinical relevance, as it compromises the effectiveness of both empirically selected regimens and therapies guided by antimicrobial susceptibility testing [26].

In our study, the most frequent co-isolation involved *E. coli* and *S. pseudintermedius* (48.2%), differing from the findings of Rosales et al. [32], where the most common combination was *S. pseudintermedius* and *M. pachydermatis* (31.8%). This discrepancy likely reflects the fact that our analysis focused exclusively on species associated with *E. coli*, *P. mirabilis*, and the *E. cloacae* complex, excluding co-infections that did not involve these bacteria.

The term “co-isolation” refers strictly to laboratory detection of multiple species in the same sample and does not necessarily imply clinical co-infection.

### 3.10. Strengths

Comprehensive antimicrobial susceptibility testing, combined with screening for ESBL and AmpC β-lactamase production, provided detailed insights into MDR and AMR in *E. coli*, *P. mirabilis*, and the *E. cloacae* complex. Assessment of co-infections with other common ear pathogens underscores the clinical relevance and zoonotic potential of resistant strains in companion animals.

### 3.11. Limitations

The study is geographically limited, which may restrict the generalizability of the findings to other regions. Additionally, the lack of molecular characterization of resistance genes limits a deeper understanding of the underlying mechanisms of MDR and AMR. AmpC and ESBL production were inferred based on phenotypic patterns interpreted by the VITEK 2 Advanced Expert System (AES), without molecular confirmation.

The small number of *E. cloacae* complex isolates (*n* = 7) limits the statistical robustness of interspecies comparisons.

Information regarding prior antimicrobial exposure and the chronic or recurrent nature of otitis externa was not available, which may influence antimicrobial resistance patterns observed in this study.

Additionally, in vitro antimicrobial susceptibility results may not fully reflect clinical efficacy in otitis externa, where topical therapy achieves high local drug concentrations.

In addition, the role of biofilm formation in the persistence and antimicrobial tolerance of pathogens involved in otitis externa was not investigated in this study, although it represents an important factor contributing to chronic infections and treatment failure [37].

## 4. Materials and Methods

### 4.1. Study Design

Canine cases of external otitis evaluated in veterinary clinics located within Timiș County, Romania, during the 2022–2025 study period constituted the study population, from which ear canal material was collected for laboratory analysis.

### 4.2. Sample Collection

A total of 592 samples, one per dog, were obtained from dogs presenting clinical manifestations consistent with otitis externa. Specimens were collected bilaterally using sterile swabs (Copan, Brescia, Italy), which were subsequently immersed in Amies transport medium (Copan, Brescia, Italy) and refrigerated at 4 °C for a period not exceeding 24 h prior to microbiological processing.

Although material was harvested from both ear canals, the unit of analysis was defined at the individual animal level, and therefore a single sample per dog was considered for further evaluation. In situations where two isolates of *E. coli*, *P. mirabilis*, or *E. cloacae* complex displaying indistinguishable antimicrobial resistance phenotypes were recovered from the same subject, only one representative strain was retained to prevent duplication of data and the potential inflation of prevalence estimates.

In cases where different bacterial species were identified in each ear, isolates corresponding to the target species (*E. coli*, *P. mirabilis*, or the *E. cloacae* complex) were prioritized for inclusion in the analysis.

Furthermore, there were cases in which *E. coli*, *P. mirabilis*, or the *E. cloacae* complex was detected exclusively in one ear, whereas the contralateral sample either yielded no bacterial growth or a different bacterial species. This methodological approach was implemented to ensure analytical consistency and to minimize bias associated with intra-individual isolate redundancy.

Co-isolation was defined as the recovery of two or more distinct microbial species from the same clinical sample.

The unit of analysis for prevalence calculations was the individual animal (*n* = 592 dogs).

### 4.3. Bacterial Isolation and Identification

Primary isolation was performed by inoculating samples onto Columbia agar enriched with 5% sheep blood (Oxoid, Basingstoke, UK) and MacConkey agar plates (Oxoid, Basingstoke, UK), followed by aerobic incubation at 37 °C for 18–24 h. Colony morphology, Gram staining, and preliminary biochemical characteristics were assessed prior to MALDI-TOF MS (Matrix-assisted laser desorption/ionization time-of-flight mass spectrometry, Bruker Daltonik, Bremen, Germany) confirmation.

For *E. coli*, presumptive identification was based on the presence of medium-sized, round, smooth, grayish colonies on blood agar and lactose-fermenting pink colonies on MacConkey agar. Gram staining revealed Gram-negative rods. Preliminary biochemical screening included oxidase negativity and positive indole production, supporting presumptive identification prior to MALDI-TOF confirmation.

*P. mirabilis* isolates were initially recognized by their characteristic swarming motility on blood agar and the formation of non-lactose-fermenting, pale colonies on MacConkey agar. Gram staining demonstrated Gram-negative bacilli. Preliminary biochemical testing included oxidase negativity, urease positivity, and phenylalanine deaminase activity, consistent with *Proteus* spp.

Members of the *E. cloacae* complex were presumptively identified by the presence of moist, gray colonies on blood agar and lactose-fermenting colonies on MacConkey agar, typically less intensely pigmented than those of *E. coli.* Gram staining confirmed Gram-negative rods. Initial biochemical characterization included oxidase negativity and variable motility, with further differentiation achieved through automated identification and MALDI-TOF confirmation due to the taxonomic complexity of the *E. cloacae* complex.

Prior to antimicrobial susceptibility evaluation, isolates presumptively identified as *E. coli*, *P. mirabilis*, or the *E. cloacae* complex were taxonomically confirmed by means of MALDI-TOF mass spectrometric analysis (Bruker Daltonik, Bremen, Germany), a matrix-assisted laser desorption and ionization time-of-flight–based platform. Bacterial protein extraction was performed using an ethanol–formic acid preparation protocol to ensure optimal spectral quality. For analysis, 1 μL of the resulting protein extract was spotted onto a designated MALDI steel target (Bruker Daltonik, Bremen, Germany), after which an equivalent volume of matrix reagent was overlaid. The matrix consisted of α-cyano-4-hydroxycinnamic acid at a concentration of 10 mg/mL, subsequently dissolved in a solvent system containing 50% acetonitrile supplemented with 2.5% trifluoroacetic acid. Spectral data acquisition was carried out using a Microflex™ mass spectrometry platform (Bruker Daltonik, Bremen, Germany), and the generated profiles were interpreted with the MALDI BioTyper™ 3.0 software suite (Bruker Daltonik, Bremen, Germany, version 3.0). Taxonomic assignment relied on comparison of the obtained peptide mass fingerprints with entries contained in the manufacturer’s reference library. Interpretation of identification scores followed the criteria recommended by Bruker: values equal to or exceeding 2.0 were regarded as consistent with species-level identification, with scores between 1.7 and 1.99 supporting identification restricted to the genus level [38].

For quality control (QC), each MALDI-TOF session was calibrated using the Bacterial Test Standard (BTS, Bruker Daltonik, Bremen, Germany) according to the manufacturer’s instructions. QC strains included *Escherichia coli* ATCC 8739 (LGC Standards, Teddington, UK) for calibration and species ID validation, *Proteus mirabilis* ATCC 29906 (LGC Standards, Teddington, UK) as a QC strain for identification, and *Enterobacter cloacae* ATCC 13047 (LGC Standards, Teddington, UK) as a QC strain for *Enterobacter* identification.

All additional bacterial and fungal species recovered from the samples (e.g., *S. pseudintermedius*, *P. aeruginosa*, *M. pachydermatis*, *S. schleiferi*) were identified using the same MALDI-TOF MS workflow described above.

### 4.4. Antimicrobial Susceptibility Testing (AST)

Antimicrobial susceptibility testing was undertaken using the integrated VITEK^®^ 2 Compact system (bioMérieux, Marcy-l’Étoile, France, version 9.02), in compliance with the manufacturer’s instructions. Prior to analysis, pure bacterial isolates were recovered, and standardized inocula were prepared by suspending colonies in sterile saline solution. The turbidity of each suspension was adjusted to attain the 0.5 McFarland standard. Cards designed for Gram-negative bacteria were used for antimicrobial susceptibility determination [39,40].

A total of 15 antimicrobial agents, categorized into six distinct antimicrobial classes, were included in the susceptibility assessment, as detailed below: ampicillin, amoxicillin–clavulanic acid, and piperacillin–tazobactam (β-lactams); cephalothin, cefuroxime, cefoxitin, cefpodoxime, cefotaxime, and ceftazidime (cephalosporins); imipenem and meropenem (carbapenems); gentamicin and tobramycin (aminoglycosides); ciprofloxacin (fluoroquinolones); and trimethoprim–sulfamethoxazole (sulfonamides + pyrimidines).

All AST procedures, interpretation of results, and QC were performed according to CLSI 2018 VET01S guidelines (CLSI, Wayne, PA, USA). Results were classified as Susceptible (S), Intermediate (I), or Resistant (R) using the species-specific breakpoints recommended for veterinary isolates [39].

Intermediate (I) results were reported separately and were not automatically classified as resistant. Based on these interpretations, MDR was defined according to the criteria proposed by Magiorakos et al. as resistance to at least one antimicrobial agent in three or more antimicrobial classes. Therefore, only isolates categorized as resistant (R) were included in the MDR analysis [41].

Resistance at the antimicrobial class level was determined according to standard criteria: an isolate was considered resistant to a given antimicrobial class if it exhibited resistance to at least one antimicrobial agent within that class, in accordance with CLSI (Clinical and Laboratory Standards Institute) guidelines.

QC strains were included in every run: *E. coli* ATCC 25922 (LGC Standards, Teddington, UK) (general QC), *Klebsiella pneumoniae* ATCC 700603 (LGC Standards, Teddington, UK) (ESBL positive control), and *Enterobacter cloacae* ATCC 13047 (AmpC positive control), to ensure the accuracy and reproducibility of AST results.

### 4.5. Detection of ESBL and AmpC β-Lactamase Production

#### 4.5.1. ESBL Screening

Screening for ESBL production was performed for all *E. coli* and *P. mirabilis* isolates using the VITEK^®^ 2 system (bioMérieux, Marcy-l’Étoile, France, version 9.02) with Gram-negative AST cards containing ceftazidime, cefotaxime, and cefpodoxime, according to CLSI 2018 guidelines [39]. Isolates showing resistance or intermediate susceptibility to these cephalosporins were considered potential ESBL producers.

#### 4.5.2. ESBL Confirmation

Confirmation of ESBL production was carried out using the VITEK^®^ 2 AES, which automatically detects ESBL phenotypes based on changes in MIC values in the presence of β-lactamase inhibitors (clavulanic acid), as per CLSI 2018 recommendations [39]. *E. coli* ATCC^®^ 25922 was used as the negative QC strain, and *K. pneumoniae* ATCC^®^ 700603 was used as the positive QC strain for ESBL detection.

#### 4.5.3. AmpC Screening

Screening for AmpC β-lactamase production was performed for all *E. cloacae* complex isolates using VITEK^®^ 2 AST results, with attention to cefoxitin resistance or reduced susceptibility, which indicates potential AmpC production [39].

#### 4.5.4. AmpC Confirmation

Confirmation of AmpC production was performed using VITEK^®^ 2 AES, which interprets MIC patterns according to CLSI 2018 guidelines to identify AmpC-producing isolates [39]. *E. coli* ATCC^®^ 25922 was used as a negative control, and *E. coli* ATCC^®^ 35218 (LGC Standards, Teddington, UK) was included as a positive β-lactamase-producing control for AmpC detection.

### 4.6. Ethical Approval

The present study was approved by the Bioethics Commission of the University of Life Sciences “King Mihai I” in Timișoara (Approval No. 644, dated 19 February 2026). The research did not involve experimental procedures performed on live animals. All activities were conducted in compliance with applicable regulations and in line with established ethical guidelines governing animal research.

### 4.7. Statistical Analysis

Comparative statistical analysis between species was performed using chi-square or Fisher’s exact test where appropriate. Ninety-five percent confidence intervals (95% CI) were calculated using the Wilson method. A *p*-value < 0.05 was considered statistically significant.

For comparisons involving small sample sizes (e.g., *E. cloacae* complex, *n* = 7), Fisher’s exact test was applied where appropriate.

## 5. Conclusions

The present study reveals that MDR and β-lactamase-producing bacteria, including *E. coli*, *P. mirabilis*, and the *E. cloacae* complex, are prevalent in dogs with otitis externa. Aminoglycoside and β-lactam resistance were notably frequent in *E. coli* and *P. mirabilis*, whereas the *E. cloacae* complex exhibited universal cephalosporin resistance but retained susceptibility to non-cephalosporin β-lactams (piperacillin–tazobactam), carbapenems and aminoglycosides. The detection of ESBL and AmpC β-lactamases underscores the potential for limited therapeutic options and highlights the need for judicious antimicrobial use. Polymicrobial infections, primarily involving *S. pseudintermedius* and *M. pachydermatis*, were common, emphasizing the complexity of otic infections in companion animals. These findings stress the importance of routine antimicrobial susceptibility testing, targeted therapy based on local resistance profiles, and continuous AMR surveillance to optimize clinical outcomes and highlight their potential public health relevance, although zoonotic transmission was not directly assessed.

## Figures and Tables

**Table 1:** Antimicrobial resistance of *E. coli*, *P. mirabilis*, and the *E. cloacae* complex isolated from dogs with otitis externa.

Antimicrobial	*E. coli*			*P. mirabilis*			*E. cloacae* Complex		
	S, *n* (%)	I, *n* (%)	R, *n* (%)	S, *n* (%)	I, *n* (%)	R, *n* (%)	S, *n* (%)	I, *n* (%)	R, *n* (%)
Ampicillin	44 (81.5)	0 (0)	10 (18.5)	17 (46.0)	0 (0)	20 (54.0)	NA	NA	NA
Amoxicillin + clavulanic acid	54 (100)	0 (0)	0 (0)	37 (100)	0 (0)	0 (0)	NA	NA	NA
Piperacillin + tazobactam	54 (100)	0 (0)	0 (0)	37 (100)	0 (0)	0 (0)	7 (100)	0 (0)	0 (0)
Cephalothin	33 (61.1)	0 (0)	21 (38.9)	34 (91.9)	0 (0)	3 (8.1)	0 (0)	0 (0)	7 (100)
Cefuroxime	33 (61.1)	14 (25.9)	7 (13)	34 (91.9)	0 (0)	3 (8.1)	0 (0)	4 (57.1)	3 (42.9)
Cefoxitin	54 (100)	0 (0)	0 (0)	37 (100)	0 (0)	0 (0)	0 (0)	0 (0)	7 (100)
Cefpodoxime	47 (87)	0 (0)	7 (13)	34 (91.9)	0 (0)	3 (8.1)	0 (0)	0 (0)	7 (100)
Cefotaxime	47 (87)	0 (0)	7 (13)	34 (91.9)	0 (0)	3 (8.1)	7 (100)	0 (0)	0 (0)
Ceftazidime	47 (87)	0 (0)	7 (13)	34 (91.9)	0 (0)	3 (8.1)	7 (100)	0 (0)	0 (0)
Imipenem	54 (100)	0 (0)	0 (0)	37 (100)	0 (0)	0 (0)	7 (100)	0 (0)	0 (0)
Meropenem	54 (100)	0 (0)	0 (0)	37 (100)	0 (0)	0 (0)	7 (100)	0 (0)	0 (0)
Gentamicin	21 (38.9)	0 (0)	33 (61.1)	31 (83.8)	0 (0)	6 (16.2)	7 (100)	0 (0)	0 (0)
Tobramycin	15 (27.8)	0 (0)	39 (72.2)	31 (83.8)	0 (0)	6 (16.2)	7 (100)	0 (0)	0 (0)
Ciprofloxacin	31 (57.4)	6 (11.1)	17 (31.5)	35 (94.6)	2 (5.4)	0 (0)	6 (85.7)	0 (0)	1 (14.3)
Trimethoprim + sulfamethoxazole	35 (64.8)	0 (0)	19 (35.2)	20 (54.0)	0 (0)	17 (46.0)	4 (57.1)	0 (0)	3 (42.9)

Legend: NA = not applicable.

**Table 2:** Distribution of antimicrobial resistance by drug class in *E. coli*, *P. mirabilis*, and the *E. cloacae* complex isolated from dogs with otitis externa.

Antimicrobial Class	*E. coli*			*P. mirabilis*			*E. cloacae* Complex		
	S, *n* (%)	I, *n* (%)	R, *n* (%)	S, *n* (%)	I, *n* (%)	R, *n* (%)	S, *n* (%)	I, *n* (%)	R, *n* (%)
Penicillins	44 (81.5)	0 (0)	10 (18.5)	17 (46.0)	0 (0)	20 (54.0)	7 (100)	0 (0)	0 (0)
Cephalosporins	33 (61.1)	0 (0)	21 (38.9)	34 (91.9)	0 (0)	3 (8.1)	0 (0)	0 (0)	7 (100)
Carbapenems	54 (100)	0 (0)	0 (0)	37 (100)	0 (0)	0 (0)	7 (100)	0 (0)	0 (0)
Aminoglycosides	15 (27.8)	0 (0)	39 (72.2)	31 (83.8)	0 (0)	6 (16.2)	7 (100)	0 (0)	0 (0)
Fluoroquinolones	31 (57.4)	6 (11.1)	17 (31.5)	35 (94.6)	2 (5.4)	0 (0)	6 (85.7)	0 (0)	1 (14.3)
Sulfonamides + Pyrimidines	35 (64.8)	0 (0)	19 (35.2)	20 (54.0)	0 (0)	17 (46.0)	4 (57.1)	0 (0)	3 (42.9)

Legend: Penicillins include ampicillin, amoxicillin–clavulanic acid, and piperacillin–tazobactam.

**Table 3:** Antimicrobial resistance of individual *E. coli* strains isolated from dogs with otitis externa.

*E. coli* (*n* = 54)	
Resistance Profile	Number of Strains and Percentage
Susceptible to all tested antibiotics	2 (3.7%)
AMP (R)	3 (5.6)
TOB (R)	2 (3.7%)
CF (R) + CFX (I)	10 (18.5%)
GEN (R) + TOB (R)	9 (16.7%)
GEN (R) + TOB (R) + CIP (R)	9 (16.7%)
GEN (R) + TOB (R) + CIP (R) + SXT (R)	8 (14.8%)
CF (R) + CFX (I) + GEN (R) + TOB (R) + CIP (I) + SXT (R)	4 (7.4%)
AMP (R) + CF (R) + CFX (R) + CPM (R) + CTX (R) + CAZ (R) + TOB (R) + SXT (R)	4 (7.4%)
AMP (R) + CF (R) + CFX (R) + CPM (R) + CTX (R) + CAZ (R) + GEN (R) + TOB (R) + CIP (I) + SXT (R)	2 (3.7%)
AMP (R) + CF (R) + CFX (R) + CPM (R) + CTX (R) + CAZ (R) + GEN (R) + TOB (R) + SXT (R)	1 (1.9%)

Legend: AMP, ampicillin; CF, cephalothin; CFX, cefuroxime; CPM, cefpodoxime; CTX, cefotaxime; CAZ, ceftazidime; GEN, gentamicin; TOB, tobramycin; CIP, ciprofloxacin; SXT, trimethoprim + sulfamethoxazole. S—susceptible, R—resistant, I—intermediate.

**Table 4:** Antimicrobial resistance of individual *P. mirabilis* strains isolated from dogs with otitis externa.

*P. mirabilis* (*n* = 37)	
Resistance Profile	Number of Strains and Percentage
Susceptible to all tested antibiotics	15 (40.5%)
AMP (R)	5 (13.5%)
SXT (R)	2 (5.4%)
AMP (R) + SXT (R)	8 (21.6%)
AMP (R) + GEN (R) + TOB (R) + SXT (R)	3 (8.1%)
AMP (R) + GEN (R) + TOB (R) + CIP (I) + SXT (R)	1 (2.7%)
AMP (R) + CF (R) + CFX (R) + CPM (R) + CTX (R) + CAZ (R) + GEN (R) + TOB (R) + SXT (R)	2 (5.4%)
AMP (R) + CF (R) + CFX (R) + CPM (R) + CTX (R) + CAZ (R) + CIP (I) + SXT (R)	1 (2.7%)

Legend: AMP, ampicillin; CF, cephalothin; CFX, cefuroxime; CPM, cefpodoxime; CTX, cefotaxime; CAZ, ceftazidime; GEN, gentamicin; TOB, tobramycin; CIP, ciprofloxacin; SXT, trimethoprim + sulfamethoxazole. S—susceptible, R—resistant, I—intermediate.

**Table 5:** Antimicrobial resistance of individual *E. cloacae* complex strains isolated from dogs with otitis externa.

*E. cloacae* Complex (*n* = 7)	
Resistance Profile	Number of Strains and Percentage
CF (R) + CFX (I) + FOX (R) + CPM (R)	4 (57.1%)
CF (R) + CFX (R) + FOX (R) + CPM (R) + SXT (R)	2 (28.6%)
CF (R) + CFX (R) + FOX (R) + CPM (R) + CIP (R) + SXT (R)	1 (14.3%)

Legend: CF, cephalothin; CFX, cefuroxime; FOX, cefoxitin; CPM, cefpodoxime; CIP, ciprofloxacin; SXT, trimethoprim + sulfamethoxazole. S—susceptible, R—resistant, I—intermediate.

**Table 7:** Species co-isolated with *E. coli* in samples from dogs with otitis externa.

Species Co-Isolated with *E. coli*	No. of Isolates, *n* (%)
None (monoinfection)	6 (11.1%)
*S. pseudintermedius*	26 (48.2%)
*M. pachydermatis*	15 (27.8%)
*P. aeruginosa*	4 (7.4%)
*S. schleiferi*	2 (3.7%)
*P. mirabilis*	1 (1.9%)

Legend: None indicates *E. coli* monoinfection (no co-isolated species).

**Table 8:** Species co-isolated with *P. mirabilis* in samples from dogs with otitis externa.

Species Co-Isolated with *P. mirabilis*	No. of Isolates, *n* (%)
None (monoinfection)	5 (13.5%)
*S. pseudintermedius*	14 (37.8%)
*M. pachydermatis*	10 (27.0%)
*P. aeruginosa*	8 (21.6%)

Legend: None indicates *P. mirabilis* monoinfection (no co-isolated species).

**Table 9:** Species co-isolated with *E. cloacae* complex in samples from dogs with otitis externa.

Species Co-Isolated with *E. cloacae* Complex	No. of Isolates, *n* (%)
None (monoinfection)	2 (28.6%)
*S. pseudintermedius*	2 (28.6%)
*M. pachydermatis*	2 (28.6%)
*P. aeruginosa*	1 (14.3%)

Legend: None indicates monoinfection (no co-isolated species).

## Data Availability

The original contributions presented in this study are included in the article. Further inquiries can be directed to the corresponding author.

## Abbreviations

The following abbreviations are used in this manuscript:

AMR	Antimicrobial resistance
*E. coli*	*Escherichia coli*
MDR	Multidrug-resistant
ESBL	Extended-spectrum beta-lactamase
*P. mirabilis*	*Proteus mirabilis*
*E. cloacae* complex	*Enterobacter cloacae* complex
AmpC	AmpC β-lactamase
MALDI-TOF MS	Matrix-assisted laser desorption/ionization time-of-flight mass spectrometry
QC	Quality control
AST	Antimicrobial Susceptibility Testing
BTS	Bacterial Test Standard
CLSI	Clinical and Laboratory Standards Institute
